# From Discovery to Manufacturing: A Quantitative Review of Phosphonates and Strategies for High-Titer Production

**DOI:** 10.3390/microorganisms14061170

**Published:** 2026-05-22

**Authors:** Xinping Zhong, Biwei Song, Lixin Zhang, Tom Hsiang, Liming Ouyang, Jingyu Zhang

**Affiliations:** 1 State Key Laboratory of Bioreactor Engineering, School of Biotechnology, East China University of Science and Technology, Shanghai 200237, China; xingping_zhong@163.com (X.Z.); yinheqilin@163.com (B.S.); lxzhang@ecust.edu.cn (L.Z.); ouyanglm@ecust.edu.cn (L.O.); 2 School of Environmental Sciences, University of Guelph, 50 Stone Road East, Guelph, ON N1G 2W1, Canada; thsiang@uoguelph.ca

**Keywords:** phosphonates, microorganisms, production improvement, genome mining

## Abstract

Phosphonate natural products, characterized by a stable carbon–phosphorus (C–P) bond, represent a distinctive class of natural products with broad bioactivities and diverse applications in medicine and agriculture. Despite their potential, the field has lacked a systematic, quantitatively informed synthesis linking discovery trends to biological function and scalable production. In this review, we curate 71 reports of phosphonate natural products between 1959 and the present, primarily from bacterial sources, and analyze them across discovery modalities, bioactivity profiles, and producer taxonomy. We further summarize structure–activity relationships and consolidate engineering strategies—including metabolic engineering, synthetic biology, and biocatalytic cascades—employed to enhance phosphonate yields. Based on these analyses, we propose practical directions to accelerate the discovery of novel phosphonates, and improve their accessibility through advances in production and isolation technologies.

## 1. Introduction

Microorganisms are a major source of natural products (NPs) and have contributed extensively to modern drug discovery [1]. Clinically important microbial metabolites include antibacterial agents such as penicillin, erythromycin, and streptomycin, anticancer agents such as doxorubicin and bleomycin, the antifungal agent amphotericin B, and the antiparasitic agent ivermectin, among others [2]. This structural and functional diversity makes microbial NPs a rich source of bioactive scaffolds. Within this reservoir, phosphonate natural products (PnNPs) are notable for their C–P bond, which confers stability and enables mimicry of metabolic substrates such as phosphate esters and carboxylates [3,4,5]. This mimicry allows phosphonates to inhibit key enzymes, leading to broad bioactivity.

**Figure 1:**
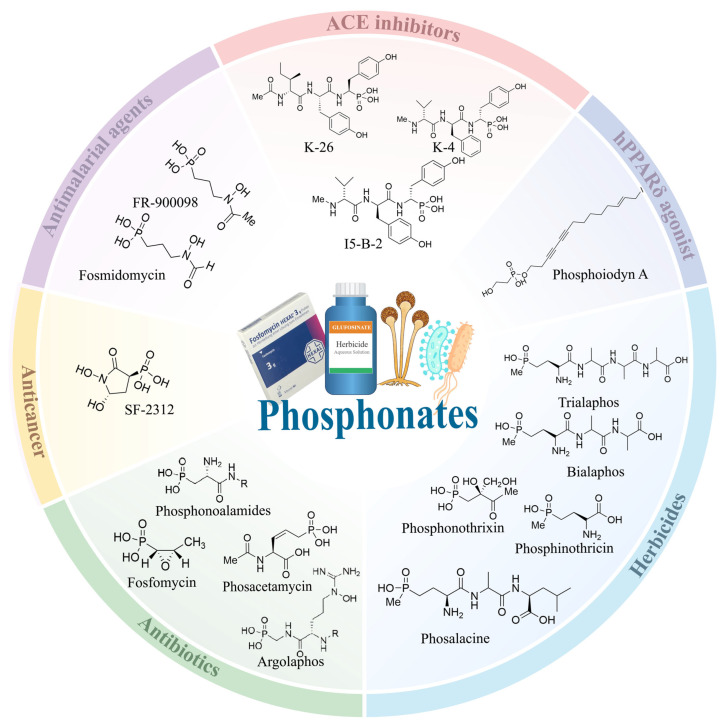
Diverse bioactivities of some phosphonates. Abbreviations: angiotensin-converting enzyme (ACE), human peroxisome proliferator-activated receptor delta (hPPARδ).

In recent years, phosphonates have emerged as valuable scaffolds in drug discovery and agrochemical development. Fosfomycin, a clinically approved phosphonate, exemplifies their therapeutic potential, exhibiting activity against multidrug-resistant pathogens such as *Escherichia coli* and *Klebsiella pneumoniae* [6,7,8]. Other phosphonates, including FR-900098 and fosmidomycin, show promise as antimalarial agents by targeting the 1-deoxy-D-xylulose 5-phosphate reductoisomerase (DXR) in the methylerythritol phosphate pathway [9,10] (Figure 1). Collectively, these examples underscore the substantial research significance of phosphonates, supporting their development as promising agents for medical and agricultural applications.

With advancements in NP chemistry, bioinformatic technology and the enrichment of genomic databases, the discovery of NPs has shifted from bioactivity-guided screening to genome mining. Genome mining involves the use of bioinformatic tools to analyze sequences and predict biosynthetic gene clusters (BGCs) that may produce targeted products [11,12]. This strategy has led to the identification of multiple phosphonates with diverse chemical structures, such as phosphonoalamides, argolaphos, pantaphos and phosphonocystoximate [13,14,15]. While studies have shown that phosphonate biosynthetic genes are diverse and abundant in nature, phosphonates that have been discovered and successfully developed for practical applications remain limited [16]. Several challenges remain in phosphonate development including the following. (i) Translation of substantial genomic potential into chemically characterized metabolites. Phosphonate biosynthetic signatures occur in ~5% of sequenced bacterial genomes, yet only 71 small molecule phosphonates have been reported, and many potential clusters are likely cryptic or poorly expressed under standard laboratory conditions [12]. (ii) Analytical “discoverability” limitations. Many phosphonates are highly polar and lack UV/Vis-active chromophores, making them easy to miss in conventional UV-guided HPLC workflows [17]. To address these bottlenecks, genetic engineering and synthetic biology have been increasingly applied to activate cryptic phosphonate BGCs and boost titers for downstream detection and isolation, via BGC activation and flux redirection [18,19,20].

While multiple review articles on phosphonates are available, a comprehensive collation coupled with quantitative analysis is still lacking, as current reviews largely focus on phosphonate biosynthetic logic [21,22,23]. In this review, we compiled 71 reports of phosphonates discovered between 1959 and 2026 (Table S1), and systematically categorized them based on discovery approaches, bioactivity, and producing strains. Furthermore, we conducted a targeted analysis of the structure-activity relationships (SARs) of phosphonates where data were available. This is also the first reported summary of the strategies employed to improve the production of phosphonates. This review highlights quantifiable synthesis in research where reported, thereby providing a reference framework for efficient discovery and development of phosphonates.

## 2. Discovery Approaches of Phosphonates

**Figure 2:**
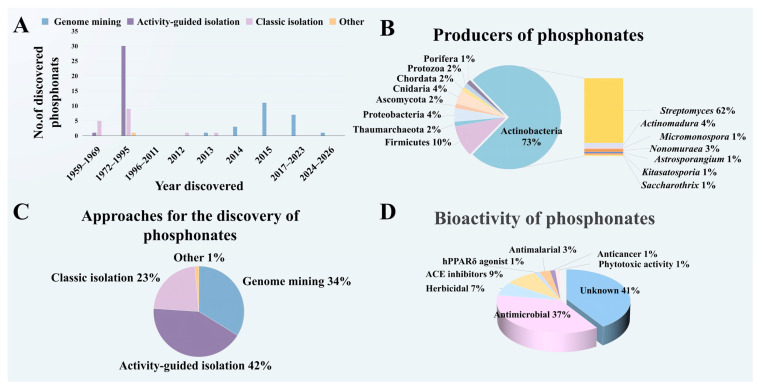
Quantitative analysis of discovery approaches, producer organisms, and bioactivities of phosphonates. (**A**) Discovery year of phosphonates; (**B**) Taxonomic distribution of phosphonate-producing organisms; (**C**) Approaches for the discovery of phosphonates; and (**D**) Bioactivity distribution of phosphonates.

The phosphonate discovery follows the historical trajectory of antibiotic discovery, having evolved through three primary phases (Figure 2C) [24,25]: (1) Activity-Guided Isolation (42%); (2) Classic Isolation (23%): classical (non-bioassay-guided) isolation and purification of compounds, with subsequent bioactivity profiling; and (3) Genome Mining (34%).

The activity-guided strategy dominated in the early discovery of phosphonates. Since the golden age of antibiotic discovery (1950s–1960s), this strategy has facilitated the identification of a wide range of NPs with promising bioactivities [24]. In 1959, researchers isolated the first phosphonate compound from the rumen of ruminants, and designated it aminoethylphosphonic acid (AEP), marking the beginning of phosphonates [26]. From 1959 to 1995, phosphonates were primarily discovered using the activity-guided isolation. Some phosphonates include fosfomycin, argolaphos, plumbemycin, fosfonochlorin, FR-900137, fosfadecin and phosacetamycin, all of which were isolated based on their antibacterial activity [14,27,28,29,30,31,32]. Additionally, rhizocticin and fosfazinomycin were isolated for their antifungal activity [33,34]. Since the discovery of phosphonothrixin in 1995, no additional phosphonates were identified for a prolonged period, resulting in a 17-year gap in the discovery of this NP scaffold [35]. This was attributed to the downturn in NP-based research and development during the 1990s, during which major pharmaceutical companies reduced their investment in the development of NP-derived drugs [36].

In the current millennium, advancements in genome sequencing technology revealed that microbes were capable of producing a much greater diversity of secondary metabolites than previously recognized [25]. This technology ushered NP discovery into a second golden age, often referred to as the genomic era. In 2012, after a 17-year hiatus, microbially derived phosphonates re-emerged in reports. Since 2013, the discovery of phosphonates has entered a new era dominated by genome mining strategy due to its advantage of high specificity, exemplified by Ju et al. (2015) using phosphoenolpyruvate (PEP) mutase as a probe to discover 11 novel phosphonates from 10,000 actinomycetes [14,21].

From 2016 to 2026, there was a slight slowdown in phosphonate discovery (Figure 2A). This trend likely reflected two factors. First, many of the previously accessible, high-yielding phosphonates had already been isolated. Second, persistent challenges in isolation, purification, and production optimization have created a long-standing bottleneck that continues to constrain phosphonate discovery.

## 3. The Producer of Phosphonates

As shown in Figure 2B, Actinobacteria (73%), particularly the genus *Streptomyces* (62%), are the primary producers of characterized phosphonates. Firmicutes (e.g., *Bacillus*) accounted for 10%, while other taxa, including Cnidaria (4%), Proteobacteria (4%), Thaumarchaeota (2%), Ascomycota (2%), Protozoa (1%), Chordata (2%), and Porifera (1%), contributed smaller proportions.

Beyond the known phosphonates, genomic data and bioinformatic analyses have revealed that the BGCs potentially producing phosphonates are widespread. For example, Wilson et al. (2023) analyzed the neighborhood distribution of genes encoding the phosphonoalanine (PnAIa) residue from the NCBI non-redundant database, and identified 95 BGCs likely to produce phosphonoalamides [13]. These BGCs were predominantly distributed in *Bacillus* (95%), with minor occurrences in *Paenibacillus* (3%), *Abyssisolibacter* (1%), and *Yanshouia* (1%). The origins of these PnAla-encoding strains were diverse, including soils, foods, plants, freshwater, marine, animals and air. Yu et al. (2013) systematically screened global microbial genomic and metagenomic datasets, including the Integrated Microbial Genomes (IMG) database, Global Ocean Sampling (GOS) marine metagenomes, and the Integrated Microbial Genomes with Microbiome Samples (IMG/M) database, using the phosphoenolpyruvate mutase gene (*pepM*) as a molecular marker [16]. Potential phosphonate producers are found in 5.7% of bacteria, 1.1% of archaea, 4.6% of soil actinomycetes, and 4.2% of eukaryotes. Additionally, the *pepM* gene was discovered in 59 samples across 79 sample sites within the GOS marine metagenomes. And 558 samples containing *pepM* genes were found in 1281 ecological samples from the IMG/M database, with the highest abundance of *pepM* genes observed in animal-associated microbiomes.

Taken together, phosphonate biosynthesis appears to be both diverse and relatively common in nature. While *Streptomyces* sp. remains the best-known source of characterized phosphonates, *Bacillus* sp. are also established producers of phosphonate scaffolds (e.g., the rhizocticins from *Bacillus subtilis* ATCC 6633) [33]. Phosphonates have additionally been documented in fungi (e.g., fosfonochlorin from *Fusarium avenaceum*), underscoring the phylogenetic breadth of this chemistry [29]. Importantly, *pepM*-centered surveys reveal extensive biosynthetic diversity beyond currently known compounds, suggesting that significant untapped potential for novel phosphonate discovery [16].

## 4. Bioactivity and Structure-Activity Relationships

Phosphonates exhibit diverse bioactivities (Figure 2D): (1) antimicrobial (37%); (2) angiotensin-converting enzyme (ACE) inhibition (9%); (3) herbicidal (7%); and (4) other activities: including antimalarial (3%), anticancer (1%), phytotoxic activity (1%) and human peroxisome proliferator-activated receptor delta (hPPARδ) agonist (1%). Notably, 41% of phosphonates have unknown activities due to low yields and hence inability to further characterize them [14,37,38,39,40,41,42,43,44]. For instance, phosphonocystoximate was discovered in 2015, but even 10 years later, its mode of action is yet reported. Although this compound had a unique structure, consisting of the S-alkyl thiohydroximate and N-acetyl-Cys moieties, whose structural features imply potential bioactivity, its low level of production has hindered bioactivity testing of the purified compound [14]. Phosphonates exert bioactivity by mimicking the natural substrate of an enzyme, and competitively binding to substrate-binding sites. Related phosphonates are presented below (Table 1).

### 4.1. Antibacterial Phosphonates

Fosfomycin (Monurol) was first isolated by Hendlin et al. (1969) from *Streptomyces fradiae* [45]. It irreversibly inhibits UDP-N-acetylglucosamine enolpyruvyl transferase (MurA) in peptidoglycan synthesis as a PEP analog. The minimum inhibitory concentrations (MIC) of fosfomycin against *E. coli* and ESBL *K. pneumoniae* are 0.5 mg/L and 4 mg/L, respectively [46]. It was among the earliest phosphonates reported to exhibit antibacterial activity.

Dehydrophos (A53868), a broad-spectrum antibacterial phosphonopeptide, was first isolated from *Streptomyces luridus* [47]. The administration of dehydrophos at a dose of 60 mg/kg significantly reduces mortality in chickens infected with *Salmonella typhimurium* [47]. Following cellular uptake, dehydrophos is hydrolyzed by peptidases to generate a C-terminal dehydroalanylmethylphosphonate moiety, which can subsequently undergo tautomerization and hydrolysis to produce methyl acetylphosphonate. This metabolite inhibits multiple pyruvate-utilizing enzymes, including pyruvate dehydrogenase, pyruvate oxidase, and 1-deoxy-D-xylulose 5-phosphate synthase (DXS) [48,49,50].

Park et al. (1976) reported a phosphono-amino-acid residue from plumbemycin (N-1409), and elucidated its structure as D-2-amino-5-phosphono-3-pentenoic acid (D-APPA) [51,52]. Subsequently, Rapp et al. identified the corresponding L-enantiomer (L-APPA) as the C-terminal residue of rhizocticin A, a family of antifungal phosphono-oligopeptides derived from *Bacillus subtilis* ATCC 6633 [53]. Based on the above research, Kugler et al. (1990) proposed a prodrug model in which rhizocticin A was intracellularly hydrolyzed by peptidases to release arginine and L-APPA, suggesting that antifungal activity was L-APPA-dependent [33]. Rhizocticin A exhibited a MIC value of 0.35 μg/mL against both *Saccharomyces cerevisiae* (Tu 125) and *Schizosaccharomyces lipolytica* (Tu 8097). This hypothesis is further supported by Laber et al., who demonstrated that APPA targets threonine synthase as an analog of homoserine phosphate, thereby determining APPA to be the key pharmacophore of these peptide NPs [54].

Flavophos (2,4-dioxopentylphosphonic acid) is a recently identified antibacterial phosphonate produced by *Burkholderia*. It selectively targets lumazine synthase (LS), a crucial enzyme in the riboflavin (vitamin B2) biosynthesis pathway, by acting as a substrate mimic of 3,4-dihydroxy-2-butanone 4-phosphate (DHBP) [55].

### 4.2. Herbicidal Phosphonates

Phosphinothricin (PPT) is a non-proteinogenic amino acid and glutamate analogue. Its peptide derivatives such as bialaphos and phosalacine, and its amino acid congeners including bialaphos (BA), phosalacine (PAL) and trialaphos (TA), are the only known phosphonates characterized by C-P-C bond [22,56,57,58]. PPT and BA were isolated from *Streptomyces viridochromogenes* by Bayer et al. (1972), while Kondo et al. (1973) isolated the same compound from *Streptomyces hygroscopicus* [59,60]. Both herbicides have been commercialized. In 1984, phosalacine (PAL) was first isolated from *Kitasatosporia phosalscinea* KA-33816, followed by the isolation of trialaphos (TA) produced by *Streptomyces hygroscopicus* KSA-1285 in 1991 [61,62]. The herbicidal activity of these compounds is mainly attributed to PPT, which is the primary active component responsible for the herbicidal activity [63]. It inhibits glutamine synthetase, an enzyme crucial for maintaining pH balance within plant cells, by mimicking glutamate. Its congeners require hydrolysis by peptidases to release the active PPT.

Phosphonothrixin, a herbicidal phosphonate, was first derived from *Saccharothrix* sp. ST-888 [35]. It can inhibit the growth of both graminaceous and broadleaf weeds, and all tested plant species, such as *Salvia viridis* L., *Echinochloa frumentacea*, *Sinapis arvensis* L., *Bidens pilosa* L. and *Amaranthus retroflexus* L., exhibited chlorosis at application dosages more than 12.35 g/ha [35]. Recently, Laber et al. (2025) demonstrated that phosphonothrixin mimics D-ribose-5-phosphate and binds to the active site of 3,4-dihydroxy-2-butanone-4-phosphate synthase (DHBPS), which contributes to the biosynthesis of vitamin B2 (riboflavin) in plants [64].

### 4.3. ACE Inhibitors

Between 1986 and 1988, a series of phosphonates with ACE inhibitory activity were isolated from actinomycetes. For example, K-26 exhibits a IC_50_ of 6.7 ng/mL against ACE; K-4 inhibits ACE with an inhibition constant (Ki) of 0.18 μM; I5-B-2 shows an IC_50_ value of 0.091μM against ACE; and SF2513 A to C inhibit sACE with IC_50_ values of 5200, 100 and 35 nM, respectively [65,66]. These ACE-inhibitory phosphonates are collectively referred to as the K-26 family. ACE, a zinc-dependent metalloprotease, is responsible for converting angiotensin I into angiotensin II [67]. K-26 family phosphonates inhibit ACE by mimicking angiotensin I. Notably, the existence of the C-P bond allows these phosphonates to directly coordinate with the zinc ion at the active site of enzyme, forming a stable complex that effectively inhibits the enzyme’s catalytic function, thereby reducing the production of angiotensin II [68].

### 4.4. hPPARδ Agonist

Phosphoiodyn A, a polyacetylene compound containing phosphorus and iodine, was first isolated by Kim et al. (2013) from Korean sponge *placospongia* sp. in South Korea [69]. The compound is identified as a potent and highly selective agonist of hPPARδ, with an EC_50_ value of 23.7 nM. The C–P bond is the principal pharmacophoric element underlying its potent and selective hPPARδ agonism [70].

### 4.5. Antimalarial Phosphonates

Phosphonates with antimalarial activity include fosmidomycin (FR-31564) and FR-900098 [71,72]. Fosmidomycin was originally isolated from Streptomyces lavendulae in 1980. It inhibits *Plasmodium falciparum* strains 3D7, HB3, Dd2 and A2, with IC_50_ values of 150, 71, 170 and 150 ng/mL, respectively [73]. FR-900098, a derivative of fosmidomycin, was first isolated from *Streptomyces rubellomurinus* [71]. It inhibits *Plasmodium falciparum* strains HB3, A2 and Dd2, with IC_50_ values of 170 ± 100, 170 ± 45, and 90 ± 20 nM, respectively [10]. Both compounds target DXR by mimicking the natural substrate, deoxyxylulose phosphate.

### 4.6. Anticancer

SF-2312 was first identified as an antibiotic in 1986 [74], and its anticancer activity was subsequently demonstrated by Leonard et al. (2016) [75]. Enolase (ENO) is a key metalloenzymes in glycolysis pathway [76]. It has three isoenzymes, namely ENO1, ENO2 and ENO3 [77]. Among these, both ENO1 and ENO2 are involved in tumor metastasis [78,79]. SF-2312 inhibits recombinant human ENO1 and ENO2 with IC_50_ values of 37.9 nM and 42.5 nM, respectively [75]. It inhibits ENO by virtue of its structural similarity to the 2-phosphoglycerate.

**Table 1:** Phosphonates with known enzyme targets and the natural substrates of the targeted enzymes. Abbreviations: 2-amino-5-phosphono-3-pentenoic acid (APPA), phosphinothricin (PPT), UDP-N-acetylglucosamine enolpyruvyl transferase (MurA), phosphoenolpyruvate (PEP), bialaphos (BA), phosalacine (PAL), trialaphos (TA), 3,4-dihydroxy-2-butanone-4-phosphate synthase (DHBPS), angiotensin-converting enzyme (ACE), human peroxisome proliferator-activated receptor delta (hPPARδ), 1-deoxy-D-xylulose 5-phosphate reductoisomerase (DXR) and enolase (ENO).

Phosphonate	Structure	Target	Natural Substrate of the Enzyme	Structure of the Natural Substrate	Reference
Fosfomycin	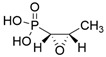	MurA	PEP	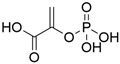	[80]
Dehydrophos	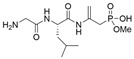	Pyruvate -utilizing enzymes	Methyl acetylphosphonate	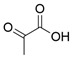	[81]
Flavophos	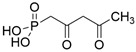	Lumazine synthase	3,4-dihydroxy-2-butanone 4-phosphate	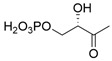	[55]
APPA	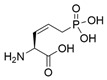	Threonine synthase	Homoserine phosphate	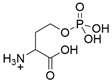	[82]
PPT	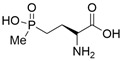	Glutamate synthetase	Glutamate	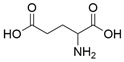	[22]
BA	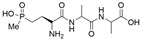				
PAL	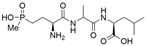				
TA	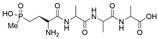				
Phosphonothrixin	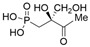	DHBPS	D-ribose-5 -phosphate	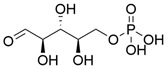	[64]
K-26	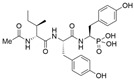	ACE	Angiotensin I	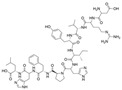	[83]
K-4	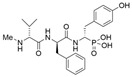				
I5-B-2	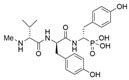				
SF2513 A	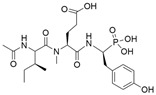				
SF2513 B	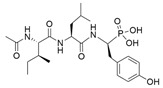				
SF2513 C	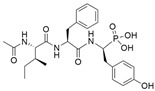				
Phosphoiodyn A	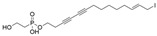	hPPARδ transcription factor	—	—	[70]
Fosmidomycin	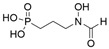	DXR	Deoxyxylulose phosphate	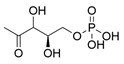	[84]
FR-900098	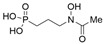				[10]
SF-2312	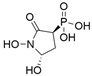	ENO	2-phosphoglycerate	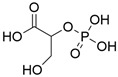	[75]

### 4.7. The Structure and Activity Relationships (SARs) of Some Phosphonates

With the growing number of phosphonates identified, studies reveal that bioactivity often depends on specific structural motifs, including phosphonate functionality, key phosphono-amino acid-derived pharmacophores (e.g., AHEP and L-PnAla), and peptide scaffolds bearing diverse side-chain and terminal modifications. In this section, we systematically summarized and illustrated the SARs of PnNPs with reported bioactivities that share the same structural skeleton, based on published literature data, with particular emphasis on the core pharmacophores proposed within their molecular architectures.

#### 4.7.1. K-26 Family

**Figure 3:**
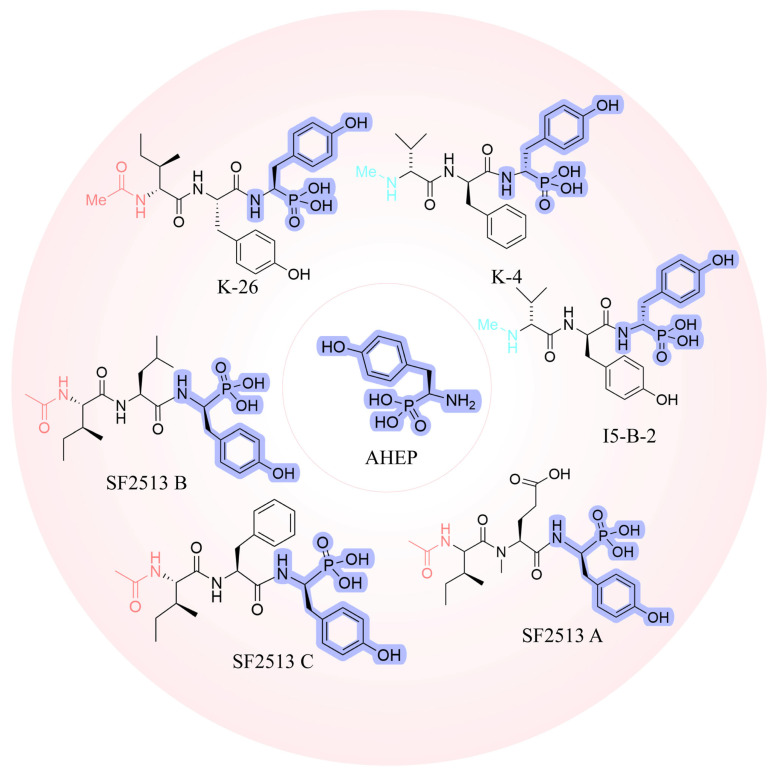
The structures of K-26 family. Abbreviation: 1-amino-2-(4-hydroxyphenyl)-ethylphosphonic acid (AHEP).

Phosphonates from K-26 family exhibit potent inhibitory ability against ACE, including K-26, K-4, SF2513 A, SF2513 B, SF2513 C and I5-B-2 [85,86]. To rationalize the determinants of this potent ACE inhibition, we detail the SARs within the K-26 family NPs as follows (Figure 3).

Firstly, 1-amino-2-(4-hydroxyphenyl)-ethylphosphonic acid (AHEP), is a key pharmacophore of the K-26 family. Its chemical structure consists of a phosphonic acid group and an aromatic phenyl ring. The phosphonic acid group directly coordinates with the zinc ion (Zn^2+^) at the active site of ACE, while simultaneously forming a hydrogen bond with conserved amino acid residues in both domains, thereby effectively inhibiting ACE activity. Additionally, the aromatic phenyl ring interacts with hydrophobic residues in the ACE active site, further enhancing the binding affinity of AHEP to ACE. Interestingly, Ntai et al. (2008) observed that the inhibitory activity of K-26 and its analogues terminated by the naturally occurring (R)-AHEP amino acid was significantly higher than that terminated by (S)-AHEP, which is consistent with the results reported by Kramer et al. (2014) [83,87].

Secondly, the N-terminal modifications of the compounds of the K-26 family has a significant impact on their ACE inhibitory activity. Kramer et al. (2014) discovered that the N-acetylated derivatives, such as K-26 and SF2513 C, exhibit stronger ACE inhibitory activity, compared to the N-methylated compounds, including K-4, SF2513 B, and I5-B-2 [83]. This is because the N-acetyl group forms hydrogen bonds with specific residues of ACE, thereby enhancing the binding to ACE and improving the inhibitory effect [68]. Additionally, the K-26 family exhibits selectivity for the N- and C-domains of ACE. The N-acetylated compounds exhibit notable C-domain selectivity. Crystallographic analyses show that the N-acetylated derivatives, K-26 and SF2513 C, preferentially bind to the C-domain of ACE rather than the N-domain, while SF2513 B displays a mild preference for the C-domain. In contrast, the N-methylated analogs, K-4 and I5-B-2, show no significant domain selectivity. Previous studies had indicated that the selectivity of ACE inhibitors for the N- and C-domains is influenced by structural variations in the amino acids within the active site. Selective inhibitors often target non-conserved amino acids between the domains and specifically interact with residues located in the S2 and S2’ sub-sites of the ACE catalytic site [88].

Finally, the inhibitory effect of the K-26 family is dependent not only on direct hydrogen bonding and hydrophobic interactions, but also involves water-mediated interactions. Crystallographic studies show that K-26 is stably bound in the binding pockets of both the C-domain and N-domain of ACE, interacting with several water molecules [83]. These water molecules might be essential in stabilizing the interaction between K-26 and ACE.

#### 4.7.2. Phosphonoalamindes

**Figure 4:**
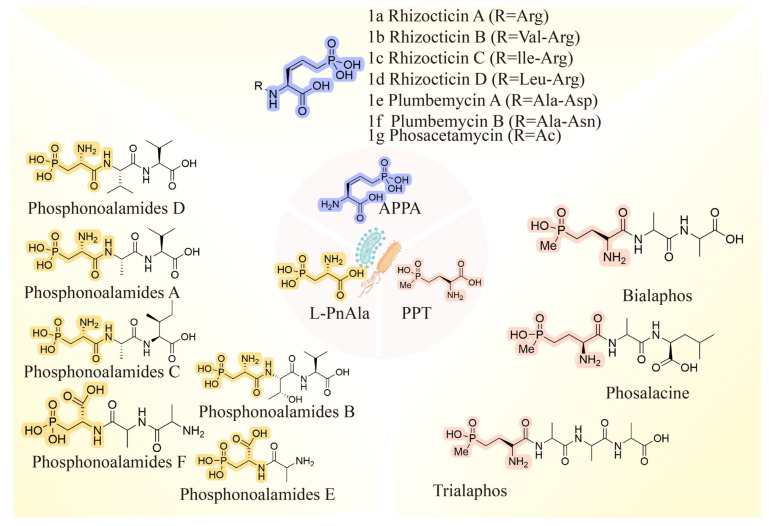
Proposed pharmacophores of some phosphonopeptides and their structures, including phosphonoalamide, rhizocticin, plumbemycin, and phosphinothricin and its amino acid derivatives. Abbreviation: L-phosphonoalanine (L-PnAla).

Phosphonoalamides are a class of phosphonopeptides [89]. Six phosphonoalamide congeners have been identified, such as phosphonoalamide A to F. Phosphonoalamide A, E, and F have demonstrated antibacterial activity, while the remaining were not tested because of low yield. The relationship between the structure and activities of those compounds (Figure 4) is summarized below.

L-PnAla, a key pharmacophore in phosphonoalamides, occurs as a constituent amino acid residue within oligopeptides. Due to their peptide-like structures, phosphonoalamides can be recognised and transported by bacterial oligopeptide transport systems, including dipeptide permease (Dpp), oligopeptide permease (Opp) and di-/tripeptide permease (Dtp) [90]. Upon entry into the cytoplasm, the peptide bonds within phosphonoalamides are hydrolyzed by bacterial cytoplasmic peptidases, leading to the release of L-PnAla as the active component. This uptake-and-release process resembles a “Trojan horse” strategy, in which peptide conjugation facilitates entry, and intracellular processing triggers activation [91].

Moreover, the amino acid residue composition can significantly affect the antibacterial activity of phosphonoalamides. For instance, Kayrouz et al. (2020) found that the antibacterial activity of phosphonoalamide A was greater than L-PnAla, which might be due to the differences in the mechanisms of cellular uptake of free phosphonic acids and phosphonopeptides [92]. Wilson et al. compared the activity differences between phosphonoalamide A (L-PnAl–L-Ala–L-Val) and phosphonoalamide F (L-Ala–L-Ala–L-PnAla) [13]. They found that phosphonoalamide F showed greater inhibitory activity against the same bacteria, such as *E. coli* K-12, *Salmonella enterica* LT2, *Pseudomonas aeruginosa* K, and *Serratia marcescens* B-2544. Furthermore, they also discovered that the antibacterial activity of tripeptide phosphonoalamide F (L-Ala-L-Ala-L-PnAla) was significantly higher than that of the dipeptide phosphonoalamide E (L-Ala-L-PnAla). Specifically, against *E. coli*, the MIC of phosphonoalamide F was 6.25 μM, while phosphonoalamide E exceeded 200 μM, resulting in a 32-fold difference. We hypothesize that the markedly higher potency of phosphonoalamide F (L-Ala–L-Ala–L-PnAla) reflects more efficient bacterial uptake and/or intracellular activation relative to the corresponding dipeptide phosphonoalamide E (L-Ala–L-PnAla).

Accordingly, these findings highlight the inherent selectivity of different oligopeptide transporters in identifying different phosphonopeptides and enabling their translocation across the bacterial cell membrane. In addition to phosphonoalamide compounds, the peptide moiety of other phosphonopeptides also may have the ‘Trojan-horse’ mechanism, such as plumbemycin (**1**e–f), phosacetamycin (**1**g) and rhizocticin (**1**a–d) [29,30,91,93]. They all also release an active molecule, APPA (Figure 3). Additionally, the amino acid derivative of phosphinothricin was found to release PPT.

#### 4.7.3. Amino Acid Congeners of PPT

Similar to phosphonoalamides, the differences in activity among congeners of PPT are also related to the amino acid residue composition and the length of the peptide chain. The amino acid congeners of PPT, including TA, BA and PAL, all contain L-PPT as the N-terminal residue. As shown in Figure 4, BA and PAL are tripeptides, differing only in the final C-terminal amino acid, which is L-alanine in BA and L-leucine in PAL. By contrast, TA is a tetrapeptide with three C-terminal alanine residues. These structural differences are accompanied by distinct bioactivity profiles. Herbicidal activity has been reported for PAL and TA, with effective concentrations of 10 μg/mL and 500 μg/mL, respectively [62,94,95]. In addition, Kato et al. evaluated the antibacterial activity of TA, BA and PAL against *E. coli* and found that BA and PAL exhibited stronger inhibitory activity than TA [96]. Among these derivatives, BA exhibits the strongest bioactivity. We speculate that BA, due to its shorter peptide chain, and the greater preference of oligopeptide transporters for its amino acid composition, exhibits superior bioactivity [97].

## 5. Strategies for Improving the Production of Phosphonates

Most phosphonates are produced at low titer in their native hosts, prompting substantial efforts to enhance their production. The strategies employed to improve production primarily focus on the following areas: (1) metabolic engineering; (2) synthetic biology; and (3) biocatalytic cascade. The following section summarizes some examples of each approach, with key studies and outcomes compiled in Table 2.

### 5.1. Metabolic Engineering

Metabolic engineering is the rational design and the genetic modification of intracellular metabolic pathways to improve the titer of target NPs [100]. The expression of key enzymes for precursor supply and downstream tailoring is often rate limiting [101]. Therefore, optimization of precursor availability and flux balance is critical. The synthesis of FR-900098 is primarily conducted through organic synthesis, and there is a lack of available green biosynthetic approaches for its production. Consequently, improving its production using biomanufacturing is essential for sustainable production.

**Figure 5:**
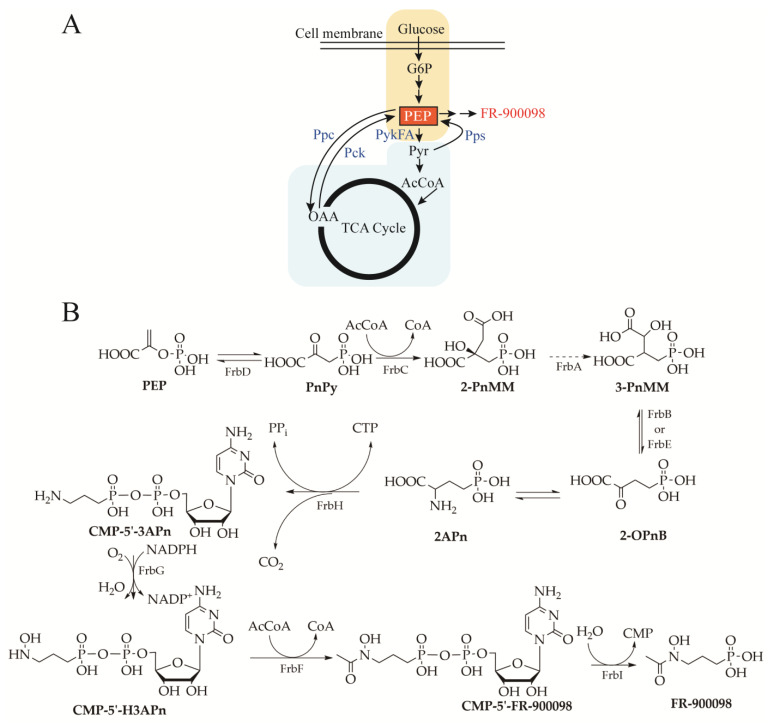
Biosynthetic logic of FR-900098. (**A**) Primary metabolic pathway for FR-900098 biosynthesis. (**B**) The biosynthetic pathway of FR-900098 [98,102]. Abbreviations: Glucose-6-phosphate (G6P), pyruvate kinase (PykFA), phosphoenolpyruvate carboxylase (Ppc), phosphoenolpyruvatesynthase (Pps), pyruvate (Pyr), oxaloacetate (OAA), acetyl-CoA (AcCoA), tricarboxylic acid (TCA), phosphonopyruvate (PnPy), 2-phosphonomethylmalate (2-PnMM), 3-phosphonomethylmalate (3-PnMM), 2-oxo-4-phosphonobutyrate (2-OPnB), 2-amino-4-phosphonobutyrate (2APn), cytidine-5′-monophosphate (CMP), CMP-5′-3-aminopropylphosphonate (CMP-5′-3APn), CMP-5′-N-hydroxy-3-aminopropylphosphonate (CMP-5′-H3APn).

PEP, a precursor of FR-900098, is metabolized through multiple enzymatic pathways. The metabolic flux distribution at the PEP node is partitioned: one branch supports FR-900098 biosynthesis, while the other branches are routed through pyruvate kinase (PykFA) and phosphoenolpyruvate carboxylase (Ppc) to generate pyruvate (Pyr) and oxaloacetate (OAA), respectively. Pyr can either contribute to FR-900098 biosynthesis via the phosphoenolpyruvatesynthase (Pps) enzyme or be further converted into acetyl-CoA (AcCoA), which enters the tricarboxylic acid (TCA) cycle. Additionally, PEP can be replenished through the conversion of OAA from the TCA cycle via phosphoenolpyruvate carboxykinase (Pck) (Figure 5A). Based on this, Desieno et al. (2012) initially attempted to improve FR-900098 production by enhancing the PEP pool via overexpressing Pck and Pps, and deleting the global regulator CsrA; however, the expected yield increase was not achieved [102]. This was because the biosynthetic network of phosphonate is complex, involving multiple key enzymes and regulatory elements. Overexpression of a single gene alone might fail to effectively improve production, and may even lead to a decrease in production caused by metabolic imbalance. With the systemic organization of metabolic networks in mind, combinatorial pathway engineering to coordinately regulate multiple pathways might address these problems. Freestone et al. (2016) adopted a combinatorial, pathway-level balancing approach [98,103]. Specifically, four promoters spanning a range of strengths (the wild-type T7 promoter and mutant derivatives) are used to titrate nine key genes (FrbA-I) in the FR-900098 pathway (Figure 5B). As a result, the heterologous expression production of FR-900098 in *E. coli* is significantly increased from 6.3 mg/L to 96 mg/L, highlighting that coordinated multi-gene regulation to rebalance metabolic flux is a key lever for improving phosphonate production.

### 5.2. Synthetic Biology

Reconstruction of the biosynthetic pathways for NPs using synthetic biology offers an effective solution to obtain bioactive NPs, whose production is otherwise constrained by exceptionally low native titer. Aminomethylphosphonate (AMP) serves as an essential intermediate in the production of glufosinate. Chu et al. (2022) refactored the AMP pathway into modular units, achieving a 500-fold increase in yield [20]. Coupled with chemical conversion, this enabled efficient glufosinate production. Specifically, they demonstrated that the alpGHIJKL genes encode enzymes required for AMP biosynthesis, which proceed through a six-step enzymatic pathway. Additionally, they discovered that the key issue behind the low AMP yield can be attributed to the low expression of alpK and alpL. Based on gene transcription levels, they divided the six genes into two modules (alpGHIJ and alpKL), and incorporated an optimized promoter–insulator–RBS regulatory triad into each module.

Accordingly, the modular division of the biosynthetic pathway together with a promoter-insulator-RBS triad enabled a precise and decoupled regulation, thereby mitigating intrinsic regulatory feedback within the native gene cluster.

### 5.3. Biocatalytic Cascade

Distinct from the approaches mentioned above, biocatalytic cascades emphasize the assembly of efficient intracellular enzyme cascades in a heterologous chassis to accomplish complex biotransformation processes [104]. This approach seems to be highly effective for enhancing the production of phosphonates.

**Figure 6:**
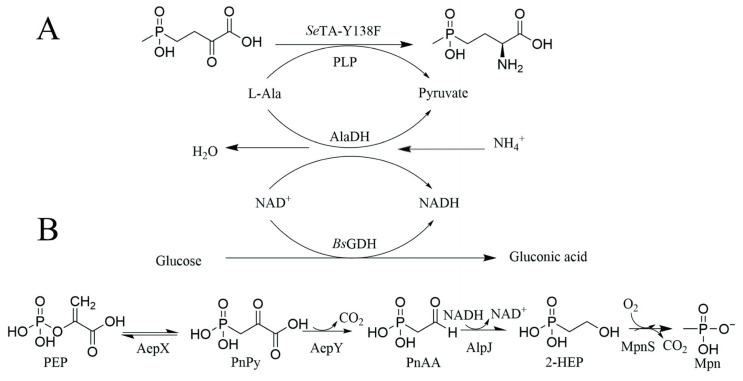
Biocatalytic cascades for enhanced phosphonate production. (**A**) A three-enzyme coupled system for biosynthesis of L-PPT; (**B**) The enzyme-catalyzed cascade for Mpn production [78,81]. Abbreviations: 4-(hydroxy(methyl) phosphoryl)-2-oxobutanoic acid (PPO), pyridoxal 5′-phosphate (PLP), L-alanine (L-Ala), *Salmonella enterica* transaminase Y138F mutant (*Se*TA-Y138F), alanine dehydrogenase (AlaDH), glucose dehydrogenase (*Bs*GDH), nicotinamide adenine ainucleotide (NAD+), reduced nicotinamide adenine ainucleotide (NADH), phosphonoacetaldehyde (PnAA) and 2-hydroxyethylphosphonate (2-HEP).

PPT is a widely used herbicide in agriculture, and its herbicidal efficacy is stereospecific, being confined to the L-enantiomer. However, commercially available herbicides are predominantly marketed as racemic mixtures of D-PPT and L-PPT [105]. Therefore, developing efficient strategies for the enantioselective production of optically pure L-PPT is a priority. Jin et al. (2022) developed a three-enzyme system to produce L-PPT, which consists of transaminase (TA), glucose dehydrogenase (BsGDH), and alanine dehydrogenase (AlaDH) in an engineered *E. coli* strain (Figure 6A) [96]. In this system, TA transfers the amino group from the amino donor to the carbonyl group of the substrate 4-(hydroxy(methyl) phosphoryl)-2-oxobutanoic acid (PPO), promoting the synthesis of L-PPT. Meanwhile, AlaDH recycles the by-product of the amino donor, reducing waste, while BsGDH regenerates the cofactor, ensuring the catalytic activity of the system is maintained. With the combined effect of three enzymes, they achieved biosynthesis of L-PPT, with a yield of 90.8% and a high enantiomeric excess of 99.9%, offering a more efficient and environmentally friendly solution for the production of L-PPT.

Methylphosphonate (Mpn) is an important intermediate in the pharmaceutical, agrochemical industries [106]. However, Mpn chemical synthesis often requires harsh conditions and generates substantial by-products. Zhang et al. (2025) are the first to construct a four-step PEP-derived enzymatic cascade, in which PEP is converted to phosphonopyruvate (PnPy), which is then transformed into phosphonoacetaldehyde (PnAA), followed by the conversion of PnAA to 2-hydrox65tyethylphosphonate (2-HEP), and ultimately to Mpn via an oxygen-dependent oxidative cleavage (Figure 6B) [99]. After in vitro optimization (AepX:AepY:AlpJ:MpnS = 1:2:2:2), the conversion of 5 mM PEP is increased by 76%. Based on the in vitro-optimized four-step enzymatic cascade reaction, the recombinant *E. coli* E6 strain is constructed as a whole-cell catalyst. Additionally, a dual-plasmid system is used, with the low-copy vector pCDFDuet expressing AepX and AepY, and the high-copy vector pETDuet expressing AlpJ and MpnS, which balance the expression of the four key enzymes. Finally, the strain produces 7.19 mM Mpn, achieving a molar conversion rate of 35.95%. This work establishes a mild aqueous “in vitro–in vivo” cooperative cascade platform by integrating cross-species enzyme selection with plasmid copy-number tuning to balance multi-enzyme expression. This provides a generalizable design paradigm for the green, scalable biosynthesis of Mpn and other complex phosphonates.

## 6. Conclusions and Prospects

Phosphonates are widely used in medicine and agriculture, and their utility is closely tied to the distinctive chemistry of the carbon–phosphorus (C–P) bond [107]. Genome-enabled discovery—especially pepM-guided approaches—has been central to the recent resurgence of phosphonate research and has accelerated pathway-led identification of candidate producers. At the same time, environmental and genomic surveys based on pepM diversity have indicated that many phosphonate biosynthetic pathways exist in nature, far beyond the number of compounds characterized to date [108]. This imbalance is consistent with the long-recognized need for tailored detection and isolation strategies to translate genomic potential into purified, structurally validated molecules.

As mentioned above, some phosphonates—especially phosphonopeptides—show similar biological activities among structurally related phosphonates, particularly phosphonopeptides, including antibacterial, herbicidal, and ACE-inhibitory activities associated with shared structural motifs, and their potency is often shaped by the accompanying co-pharmacophore (e.g., APPA, AHEP, or PPT). This relationship suggests that a dual strategy integrating bioactive building block-based cues with genome mining-driven prioritization can improve the efficiency of identifying bioactive phosphonate metabolites. In this context, combining pepM-centered genome mining with the targeted search for tailoring enzymes linked to bioactive architectures (such as NRPS modules or ATP-grasp ligases) may further facilitate the discovery of novel bioactive phosphonates. In addition, the integration of LC-MS-based screening with NMR-based metabolomics can markedly improve the accuracy, selectivity, and reliability of phosphonate detection and structural identification, particularly in complex fermentation matrices. This complementary analytical strategy will be especially valuable for overcoming the current detection challenges of low-abundance PnNPs, enabling more efficient dereplication.

Beyond target identification, a deeper SARs understanding of phosphonates will also enable the rational expansion of phosphonate chemical space. One feasible route is to produce known bioactive phosphonates biosynthetically and then derivatize them through SAR-guided chemical modification. This approach preserves the core phosphonate scaffold while allowing SAR-guided modification of peptide chain length, amino acid composition, substituent groups and other structural features relevant to bioactivity. In addition, artificial intelligence (AI)-assisted SARs modeling can prioritize high-value analogs before labor-intensive synthesis. By combining bioactivity data from both PnNPs and chemically synthesized analogues, we can significantly diversify the training dataset, which should improve the accuracy and reliability of the models. These SARs-informed, data-driven designs can then be executed through in vitro enzymatic biocatalysis and/or organic synthesis to systematically vary the phosphonate moiety, the peptide/scaffold context, and co-pharmacophore elements. Such an integrated AI–biocatalysis/organic synthesis workflow should therefore accelerate the generation of broader panels of phosphonate congeners with improved activity profiles, ultimately streamlining bioactive phosphonate discovery and development.

Next, because polarity is a recurring challenge for phosphonates, addressing polarity directly is therefore attractive. Temporary masking of phosphonate functionality via controlled esterification may offer a practical means to facilitate enrichment and separation. In addition, the rapid expansion of phosphate-binding metal–organic framework materials (MOF) suggests a plausible route toward phosphonate-selective capture media, which could improve cleanup and preconcentration from complex extracts [109].

In parallel, substantial efforts have been made to boost phosphonate titers through pathway refactoring, host engineering, and process optimization [98]. Metabolic engineering and synthetic biology enable the optimization and reconstruction of phosphonate biosynthetic pathways, but their industrial application is often limited by metabolic flux constraints, regulatory complexity, and relatively high costs associated with strain engineering and scale-up bioprocessing. In contrast, biocatalytic cascade systems provide a more controllable, potentially cost-effective, and efficient platform by decoupling synthesis from cellular regulation; however, their large-scale application still depends on improvements in enzyme stability, cofactor regeneration, and overall process economics. Collectively, these studies reinforce the view that phosphonate biosynthesis is typically governed by distributed control spanning precursor availability, multi-step enzymology, metabolite flux balance, and regulatory circuitry, rather than by a single easily amplified bottleneck. Accordingly, single-gene overexpression frequently delivers marginal gains and can be counterproductive when it perturbs pathway balance and cellular homeostasis. Productive strain improvement therefore tends to rely on coordinated multi-parameter optimization.

Notably, recent studies on dynamic and multiplexed regulation in *Streptomyces* offer useful conceptual references for future phosphonate overproduction. For example, Yang et al. (2025) developed a plug-and-play, multitarget fine-tuning paradigm, termed *Streptomyces* multiplexed artificial control system (SMARTS), and demonstrated its scalability by achieving a baiweimectin titer of 8.4 g L^−1^ in a 120 m^3^ industrial fermenter, providing an instructive case for orchestrating secondary metabolite production at scale [110]. Building on this conceptual framework, it is plausible that phosphonate overproduction could benefit from synchronized control that (i) reinforces precursor pools, (ii) throttles competing pathways, and (iii) dynamically coordinates the activities of multiple enzymes across the biosynthetic machinery, rather than relying on static, high-level expression. In addition to fully in vivo routes, hybrid manufacturing strategies—in which key intermediates are overproduced in heterologous hosts and subsequently converted to final products using downstream biocatalysts in vitro (e.g., cell-free enzymatic steps or modular cascades)—may provide alternative and potentially scalable routes to phosphonate biomanufacturing, particularly for pathways constrained by intracellular toxicity, regulation, or compartmentalized cofactor requirements.

Collectively, this review synthesizes the phosphonate field from discovery to application and production. We compile and, where possible, quantitatively summarize discovery approaches, bioactivities, and producing organisms/strains. In particular, we systematically summarize the SARs of some phosphonates and critically assess the principal strategies that have been used to enhance phosphonate production, from pathway refactoring to chassis and process optimization. Together, these perspectives aim to provide an integrated framework to accelerate the discovery, engineering, and scalable manufacture of phosphonates for therapeutic, agricultural, and broader biotechnological applications.

## Figures and Tables

**Table 2:** Reported strategies for yield improvement of phosphonates. Abbreviations: aminomethylphosphonate (AMP) and methylphosphonate (MPn).

Phosphonates	Approach	Native Strain	Heterologous Chassis	Titer	Reference
FR-900098	Metabolic engineering	*Streptomyces rubellomurinus*	*E. coli* BL21(DE3)	96 mg/L	[98]
AMP	Synthetic biology	*Streptomyces monomycini*	*Streptomyces lividans* 66	52 mg/L	[20]
L-PPT	Biocatalytic cascade	*Streptomyces hygroscopicus*	*E. coli*	A yield of 90.8%	[96]
MPn	Biocatalytic cascade	*Nitrosopumilus maritimus*	*E. coli*	A 35.95% molar conversion yield	[99]

## Data Availability

No new data were created.

## Supplementary Materials

The following supporting information can be downloaded at: https://www.mdpi.com/article/10.3390/microorganisms14061170/s1, Table S1: Phosphonate structure, bioactivity, year of discovery, producer, approach of discovery and reference.
