# Supplementary material for: From Discovery to Manufacturing: A Quantitative Review of Phosphonates and Strategies for High-Titer Production
Source: Microorganisms. 2026 May 22;14(6):1170. doi: 10.3390/microorganisms14061170 (PMC13302981; doi:10.3390/microorganisms14061170)
Supplement: Supplementary file 1 [file microorganisms-14-01170-s001.zip › microorganisms-4278293-supplementary.pdf]

Supporting Information

From Discovery to Manufacturing: A Quantitative Review of Phosphonates and Strategies for High-Titer Production

Xinping Zhong [1,†], Biwei Song [1,†], Lixin Zhang [1], Tom Hsiang [2], Liming Ouyang [1] and Jingyu Zhang [1,*]

[1] State Key Laboratory of Bioreactor Engineering, School of Biotechnology, East China University of Science and Technology, Shanghai 200237, China; xingping_zhong@163.com (X.Z.); yinheqilin@163.com (B.S.); lxzhang@ecust.edu.cn (L.Z.); ouyanglm@ecust.edu.cn (L.O.)
[2] School of Environmental Sciences, University of Guelph, 50 Stone Road East, Guelph, ON N1G 2W1, Canada; thsiang@uoguelph.ca
* Correspondence: zhangjingyu@ecust.edu.cn
† These authors contributed equally to this work.

Academic Editor: Laurent Dufossé

Received: 9 April 2026
Revised: 5 May 2026
Accepted: 16 May 2026
Published: 22 May 2026

Copyright: © 2026 by the authors. Licensee MDPI, Basel, Switzerland. This article is an open access article distributed under the terms and conditions of the Creative Commons Attribution (CC BY) license.

Contents

1. Supplementary Table 3

Table S1. Phosphonate structure, bioactivity, year of discovery, producer, approach of discovery and reference.**3**

2.REFERENCE..........**12**

1. Supplementary Table

Table S1. Phosphonate structure, bioactivity, year of discovery, producer, approach of discovery and reference.

Structure	Compound name	Bioactivity	Year discovered	Producer	Approach discovered	Reference
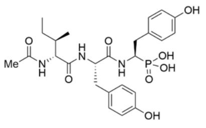	K-26	ACE inhibitor	1986	*Actinomycete* K-26	Activity-guided isolation	[1]
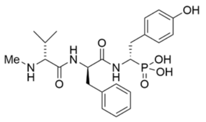	K-4	ACE inhibitor	1986	*Actinomadura* sp.	Activity-guided isolation	[2]
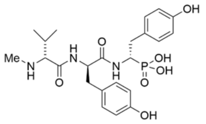	I5-B-2	ACE inhibitor	1984	*Actinomadura* sp.	Activity-guided isolation	[3]
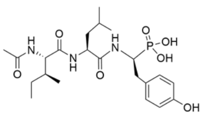	SF2513 B	ACE inhibitor	1988	*Streptosporangium nondiastaticum*	Activity-guided isolation	[4]

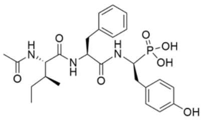	SF2513 C	ACE inhibitor	1988	*Streptosporangium nondiastaticum*	Activity-guided isolation	[4]
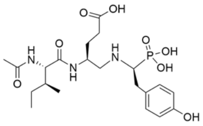	SF2513 A	ACE inhibitor	1988	*Streptosporangium nondiastaticum*	Activity-guided isolation	[4]
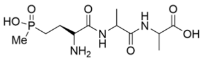	Bialaphos	Herbicidal	1972	*Streptomyces hygroscopicus* SF-1293	Activity-guided isolation	[5,6]
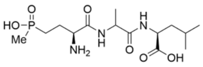	Phosalacine	Herbicidal	1984	*Kitasatospora phosalacinea* KA-338	Activity-guided isolation	[7]
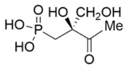	Phosphonothrixin	Herbicidal	1995	*Saccharothrix* sp. ST-888	Activity-guided isolation	[8]
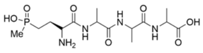	Trialaphos	Herbicidal	1991	*Streptomyces hygroscopicus* sp. KSB-1285	Activity-guided isolation	[9]
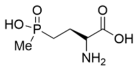	Phosphinothricin	Herbicidal	1972	*Streptomyces viridochromogene*	Activity-guided	[6]

					isolation	
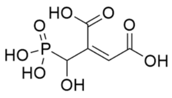	Pantaphos	Phytotoxic activity	2021	*Pantoea ananatis*	Genome mining	[10]
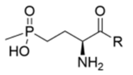 1a :R=L-Gly-L-Ala<br>1b: R=L-Ala-L-Gly<br>1c: R=L-Ala-a-amino butyric acid<br>1d: R=L-Ala-L-Val<br>1e: R=L-Ala-L-Ser<br>1f: R=L-Ala-L-Ala-PT<br>1k: R=L-Ala-L-Ala-PT-L-Ala-L-Ala	——	Unknown	1991	*Streptomyces hygroscopicus*	Classic isolation	[11]
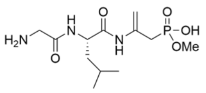	Dehydroph os	Antimicro bial	1984	*Streptomyces luridus*	Activity-guided isolation	[12]
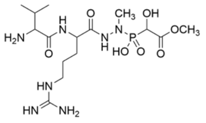	Fosfazinom ycin A	Antimicro bial	1983	*Streptomyces lavendofoliae* 630	Activity-guided isolation	[13]
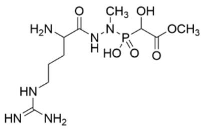	Fosfazinom ycin B	Antimicro bial	1983	*Streptomyces lavendofoliae* 630	Activity-guided isolation	[13]
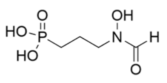	Fosmidomy cin (FR - 31564)	Antimalar ial	1980	*Streptomyces lavendulae*	Activity-guided isolation	[14]

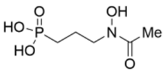	FR-900098	Antimalarial	1980	*Streptomyces rubellomurinus*	Activity-guided isolation	[15]
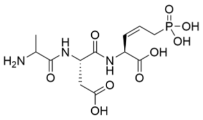	Plumbemycin A	Antimicrobial	1977	*Streptomyces plumbeus*	Activity-guided isolation	[16]
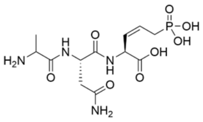	Plumbemycin B	Antimicrobial	1977	*Streptomyces plumbeus*	Activity-guided isolation	[16]
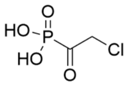	Fosfonochlorin	Antimicrobial	1989	*Fusarium avenaceum*	Activity-guided isolation	[17]
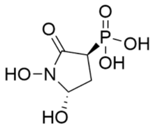	SF - 2312	Anticancer	1986	*Actinomycetes micromonospora*	Activity-guided isolation	[18]
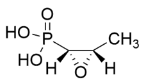	Fosfomycin	Antimicrobial	1969	*Streptomyces fradiae*	Activity-guided isolation	[19]
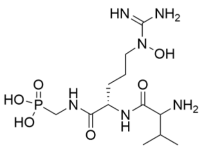	Argolaphos A	Antimicrobial	2015	*Streptomyces monomycini* NRRL	Genome mining	[20]

				B-24309		
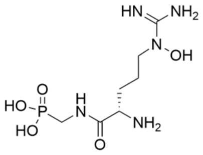	Argolaphos B	Antimicrobial	2015	*Streptomyces monomycini* NRRL B-24309	Genome mining	[20]
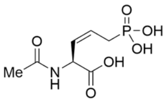	Phosacetamycin	Antimicrobial	2013	*Streptomyces aureus* NRRL B-2808	Genome mining	[21]
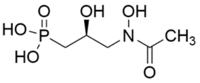	FR - 33289	Antimicrobial	1980	*Streptomyces rubellomurinus*	Activity-guided isolation	[22]
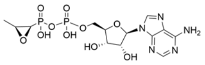	Fosfadecin	Antimicrobial	1990	*Pseudomonas viridiflava* PK-5	Activity-guided isolation	[23]
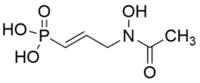	FR - 32863	Antimicrobial	1980	*Streptomyces lavendulae*	Activity-guided isolation	[24]
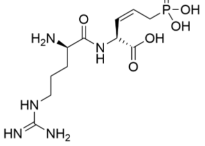	Rhizocticin A	Antimicrobial	1988	*Bacillus subtilis* ATCC 6633	Activity-guided isolation	[25]

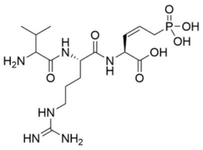	Rhizocticin B	Antimicrobial	1988	*Bacillus subtilis* ATCC 6633	Activity-guided isolation	[25]
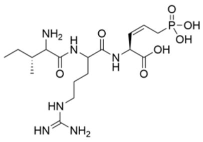	Rhizocticin C	Antimicrobial	1988	*Bacillus subtilis* ATCC 6633	Activity-guided isolation	[25]
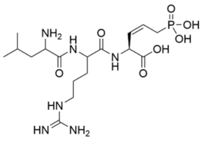	Rhizocticin D	Antimicrobial	1988	*Bacillus subtilis* ATCC 6633	Activity-guided isolation	[25]
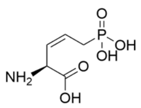	2-amino-5-phosphono-3-pentenoic acid (APPA)	Antimicrobial	1976	*Streptomyces plumbeus*	Activity-guided isolation	[26]
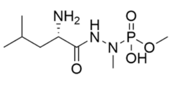	FR-900137	Antimicrobial	1980	*Streptomyces unzenensis*	Activity-guided isolation	[27]
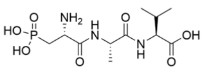	Phosphonoalamides A	Antimicrobial	2020	*Streptomyces* sp. B-2790	Genome mining	[28]

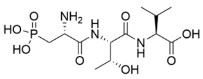	Phosphono alamides B	Unknown	2020	*Streptomyces* sp. *NRRL* B-2790	Genome mining	[28]
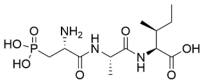	Phosphono alamides C	Unknown	2020	*Streptomyces* sp. NRRL B-2790	Genome mining	[28]
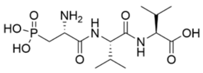	Phosphono alamides D	Unknown	2020	*Streptomyces* sp. NRRL B-2790	Genome mining	[28]
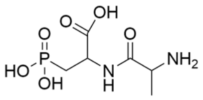	Phosphono alamides E	Antimicro bial	2023	*Bacillus velezensis*	Genome mining	[29]
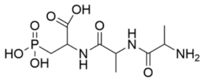	Phosphono alamides F	Antimicro bial	2023	*Bacillus velezensis*	Genome mining	[29]
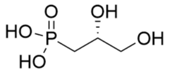	(R) - 2,3 dihydroxyp ropylphosp honic acid (DHPPA)	Antimicro bial	2015	*Streptomyces durhamensis* NRRL B-3309	Genome mining	[20]
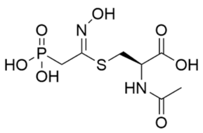	Phosphono cystoximic acid	Unknown	2015	*Streptomyces* sp. NRRL S-474 and NRRL S-481	Genome mining	[20]

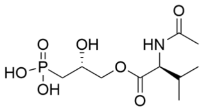	Valinophos	Unknown	2015	*Streptomyces durhamensis* NRRL B-3309	Genome mining	[20]
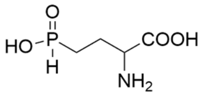	Desmethylphosphinothricin	Unknown	2015	*Nonomuraea candida* NRRL B-24552	Genome mining	[20]
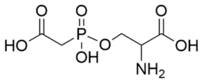	O -phosphonoacetic acid serine	Unknown	2017	*Streptomyces* NRRL F-525	Genome mining	[30]
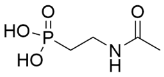	(2-acetamidoethyl)phosphonic acid	Unknown	2014	*Streptomyces regensis* WC-3744	Genome mining	[31]
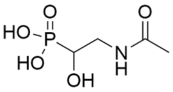	(2-acetamido-1-hydroxyethyl)phosphonic acid	Unknown	2014	*Streptomyces regensis* WC-3744	Genome mining	[31]
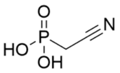	Nitrilaphos	Unknown	2015	*Streptomyces*	Genome	[20]

					mining	
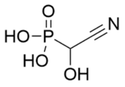	Hydroxynitrilaphos	Unknown	2014	*Streptomyces regensis* WC-3744	Genome mining	[31]
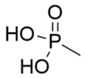	Methylphosphonic acid	Unknown	2012	*Nitrosopumilus maritimus*	Classic isolation	[32]
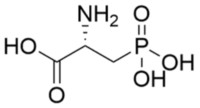	2-amino-3-phosphonopropanoic acid	Unknown	1964	*Zoanthus sociatus*	Classic isolation	[33]
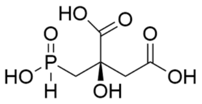	2-phosphinomethylmalic acid	Unknown	2015	*Nonomuraea candida* NRRL B-24552	Genome mining	[20]
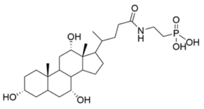	Ciliatocholic acid	Unknown	1976	Bos taurus	Classic isolation	[34]
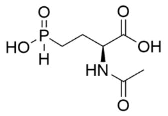	N-acetyl-demethylphosphinothricin	Unknown	1985	*Streptomyces hygroscopicus* SF-1293	Classic isolation	[35]

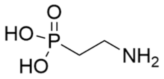	AEP	Unknown	1959	*Rumen protozoa*	Classic isolation	[36]
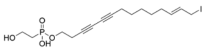	Phosphoiodyn A	hPPARδ agonist	2013	*Korean sponge placospongia* sp.	Classic isolation	[37]
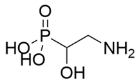	1-hydroxy-2-aminoethylphosphonate	Antimicrobial	2015	*Streptomyces* sp. NRRL S-481	Genome mining	[20]
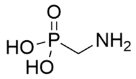	Aminomethylphosphonic acid	Antimicrobial	2015	*Streptomyces monomycini* NRRL B-24309	Genome mining	[20]
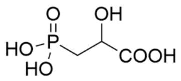	2-hydroxy-3-phosphonopropanoic acid	Unknown	2015	*Streptomyces durhamensis* NRRL B-3309	Genome mining	[20]
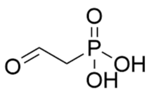	Phosphonoacetylaldehyde	Unknown	1968	*Bacillus cereus*	Classic isolation	[38]
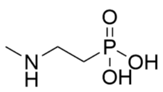	N-methylciliatine	Unknown	1967	*Anthopleura xanthogrammica*	Classic isolation	[39]

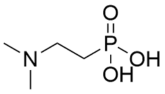	N,N-dimethylciliatine	Unknown	1967	*Anthopleura xanthogrammica*	Classic isolation	[39]
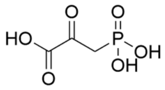	Phosphonopyruvic acid	Unknown	1990	*Streptomyces hygroscopicus* SF-1293	Other	[40]
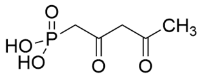	Flavophos	Antimicrobial	2026	*Burkholderia*	Genome mining	[41]

REFERENCE

1. Yamato, M.; Koguchi, T.; Okachi, R.; Yamada, K.; Nakayama, K.; Kase, H.; Karasawa, A.; Shuto, K. K-26, a novel inhibitor of angiotensin I converting enzyme produced by an *Actinomycete* K-26. *J Antibiot.* **1986**, *39*, 44–52.
2. Koguchi, T.; Yamada, K.; Yamato, M.; Okachi, R.; Nakayama, K.; Kase, H. K-4, a novel inhibitor of angiotensin I converting enzyme produced by *Actinomadura spiculosospora*. *J Antibiot.* **1986**, *39*, 364–371.
3. Kido, Y.; Hamakado, T.; Anno, M.; Miyagawa, E.; Motoki, Y.; Wakamiya, T.; Shiba, T. Isolation and characterization of I5B2, a new phosphorus containing inhibitor of angiotensin I converting enzyme produced by *Actinomadura* sp. *J Antibiot.* **1984**, *37*, 965–969.
4. Shokichi, O.; Kunihiko, K.; Akiko, S.; Tamako, T.; Junko, Y.; Shouichi, A.; Shinij, M.; Yuji, M.; Takashi, S.; Takashi, S. New angiotensin converting enzyme inhibitors SF2513 A, B and C, produced by *Streptosporangium nondiastaticum*. *Meiji Seika Kenkyu Nenpo* **1988**, *27*, 46–54.
5. Kondo, Y. Studies on a new antibiotic, SF-1293. I. Isolation and physicochemical and biological characterization of SF-1293 substances. *Sci. Rep. Meiji Seika* **1973**, *13*, 34–41.
6. Bayer, v.E.; Gugel, K.; Hägele, K.; Hagenmaier, H.; Jessipow, S.; König, W.; Zähner, H. Phosphinothricin und phosphinothricyl-alanyl-alanin. *Helv. Chim. Acta* **1972**, *55, 224-239*.
7. Omura, S.; Hinotozawa, K.; Imamura, N.; Murata, M. The structure of phosalacine, a new herbicidal antibiotic containing phosphinothricin. *J Antibiot.* **1984**, *37*, 939–940.
8. Takahashi, E.; Kimura, T.; Nakamura, K.; Arahira, M.; Iida, M. Phosphonothrixin, a novel herbicidal antibiotic produced by *Saccharothrix* sp. ST-888 I. Taxonomy, fermentation, isolation and biological properties. *J Antibiot.* **1995**, *48*, 1124–1129.
9. Kato, H.; Nagayama, K.; Abe, H.; Kobayashi, R.; Ishihara, E. Isolation, structure and biological activity of trialaphos. *Agric Biol Chem.* **1991**, *55*, 1133–1134.
10. Polidore, A.L.A.; Furiassi, L.; Hergenrother, P.J.; Metcalf, W.W. A phosphonate natural product made by pantoea ananatis is necessary and sufficient for the hallmark lesions of onion center rot. *mBio* **2021**, *12*, e03402-20.
11. Kumada, Y.; Imai, S.; Nagaoka, K. Conversion of bialaphos to other oligopeptides containing phosphinothricin by *Streptomyces hygroscopicus*. *J Antibiot.* **1991**, *44*, 1006–1012.

12. Johnson, R.D.; Kastner, R.M.; Larsen, S.H.; Ose, E.E. Antibiotic A53868 and process for production thereof. United States patent US4463092A. July 31, **1984**.
13. Ogita, T.; Gunji, S.; Fukazawa, Y.; Terahara, A.; Kinoshita, T.; Nagaki, H.; Beppu, T. The structures of fosfazinomycins A and B. *Tetrahedron Lett.* **1983**, *24*, 2283–2286.
14. Okuhara, M.; Kuroda, Y.; Goto, T.; Okamoto, M.; Terano, H.; Kohsaka, M.; Aoki, H.; Imanaka, H. Studies on new phosphonic acid antibiotics III. Isolation and characterization of FR-31564. *J Antibiot.* **1980**, *33*, 24–28.
15. Okuhara, M.; Kuroda, Y.; Goto, T.; Okamoto, M.; Terano, H.; Kohsaka, M.; Aoki, H.; Imanaka, H. Studies on new phosphonic acid antibiotics. I. FR-900098, isolation and characterization. *J Antibiot.* **1980**, *33*, 13–17.
16. Park, B.K.; Hirota, A.; Sakai, H. Studies on new antimetabolite N-1409. *Agric Biol Chem* **1977**, *41*, 161–167.
17. Takeuchi, M.; Nakajima, M.; Ogita, T.; Inukai, M.; Kodama, K.; Furuya, K.; Nagaki, H.; Haneishi, T. Fosfonochlorin, a new antibiotic with spheroplast forming activity. *J Antibiot.* **1989**, *42*, 198–205.
18. Watanabe, H.; Yoshida, J.; Tanaka, E.; Ito, M.; Miyadoh, S.; Shomura, T. Studies on a new phosphonic acid antibiotic, SF-2312. *Sci Rep Meiji Seika Kaisha* **1986**, *25*, 12–17.
19. Hendlin, D.; Stapley, E.; Jackson, M.; Wallick, H.; Miller, A.; Wolf, F.; Miller, T.; Chaiet, L.; Kahan, F.; Foltz, E. Phosphonomycin, a new antibiotic produced by strains of *streptomyces*. *Science* **1969**, *166*, 122–123
20. Ju, K.-S.; Gao, J.; Doroghazi, J.R.; Wang, K.-K.A.; Thibodeaux, C.J.; Li, S.; Metzger, E.; Fudala, J.; Su, J.; Zhang, J.K.; et al. Discovery of phosphonic acid natural products by mining the genomes of 10,000 *Actinomycetes*. *Proc Natl Acad Sci U S A.* **2015**, *112*, 12175–12180.
21. Evans, B.S.; Zhao, C.; Gao, J.; Evans, C.M.; Ju, K.-S.; Doroghazi, J.R.; Van Der Donk, W.A.; Kelleher, N.L.; Metcalf, W.W. Discovery of the antibiotic phosacetamycin via a new mass spectrometry-based method for phosphonic acid detection. *ACS Chem Biol.* **2013**, *8*, 908–913.
22. Okuhara, M.; Kuroda, Y.; Goto, T.; Okamoto, M.; Terano, H.; Kohsaka, M.; Aoki, H.; Imanaka, H. Studies on new phosphonic acid antibiotics III. isolation and characterization of FR-31564. *J Antibiot.* **1980**, *33*, 24–28.
23. Katayama, N.; Tsubotani, S.; Nozaki, Y.; Harada, S.; Ono, H. Fosfadecin and fosfocytocin, new nucleotide antibiotics produced by bacteria. *J Antibiot.* **1990**, *43*, 238–246.
24. Okuhara, M.; Kuroda, Y.; Goto, T.; Okamoto, M.; Terano, H.; Kohsaka, M.; Aoki, H.; Imanaka, H. Studies on new phosphonic acid antibiotics III. isolation and characterization of FR-31564. *J Antibiot.* **1980**, *33*, 24–28.
25. Rapp, C.; Jung, G.; Kugler, M.; Loeffler, W. Rhizocticins—New phosphono-oligopeptides with antifungal activity. *Lieb. Ann. Chem* **1988**, *1988*, 655–661.
26. Park, B.K.; Hirota, A.; Sakai, H. 2-Amino-5-phosphono-3-pentenoic acid, a new amino acid from N-1409 substance, an antagonist of threonine. Agric. Biol. Chem. 1976, 40, 1905–1906.
27. Kuroda, Y.; Tanaka, H.; Okamoto, M.; Goto, T.; Kohsaka, M.; Aoki, H.; Imanaka, H. FR-900137, a new antibiotic II. Structure determination of FR-900137. *J Antibiot.* **1980**, *33*, 280–283.
28. Kayrouz, C.M.; Zhang, Y.; Pham, T.M.; Ju, K.-S. Genome mining reveals the phosphonoalamide natural products and a new route in phosphonic acid biosynthesis. *ACS Chem Biol.* **2020**, *15*, 1921–1929.
29. Wilson, J.; Cui, J.; Nakao, T.; Kwok, H.; Zhang, Y.; Kayrouz, C.M.; Pham, T.M.; Roodhouse, H.; Ju, K.-S. Discovery of antimicrobial phosphonopeptide natural products from *Bacillus velezensis* by genome mining. *Appl Environ Microbiol* **2023**, *89*, e0033823.
30. Freestone, T.S.; Ju, K.-S.; Wang, B.; Zhao, H. Discovery of a phosphonoacetic acid derived natural product by pathway refactoring. *ACS Synth Biol.* **2017**, *6*, 217–223.
31. Cioni, J.P.; Doroghazi, J.R.; Ju, K.-S.; Yu, X.; Evans, B.S.; Lee, J.; Metcalf, W.W. Cyanohydrin phosphonate natural product from *Streptomyces regensis*. *J Nat Prod.* **2014**, *77*, 243–249.

32. Metcalf, W.W.; Griffin, B.M.; Cicchillo, R.M.; Gao, J.; Janga, S.C.; Cooke, H.A.; Circello, B.T.; Evans, B.S.; Martens-Habbena, W.; Stahl, D.A. Synthesis of methylphosphonic acid by marine microbes: a source for methane in the aerobic ocean. *Science* **2012**, *337*, 1104–1107.
33. Kittredge, J.; Hughes, R. The occurrence of α-Amino-β-phosphonopropionic acid in the Zoanthid, Zoanthus sociatus, and the ciliate, Tetrahymena pyriformis. *Biochemistry* **1964**, *3*, 991–996.
34. Tamari, M.; Ogawa, M.; Kametaka, M. A new bile acid conjugate, ciliatocholic acid, from bovine gall bladder bile. *J Biochem.* **1976**, *80*, 371–377.
35. Imai, S.; Seto, H.; Sasaki, T.; Tsuruoka, T.; Ogawa, H.; Satoh, A.; Inouye, S.; Niida, T.; Otake, N. Studies on the biosynthesis of bialaphos (SF-1293) 6. Production of N-acetyl-demethylphosphinothricin and N-acetylbialaphos by blocked mutants of *Streptomyces hygroscopicus* SF-1293 and their roles in the biosynthesis of bialaphos. *J Antibiot.* **1985**, *38*, 687–690.
36. Horiguchi, M.; Kandatsu, M. Isolation of 2-aminoethane phosphonic acid from rumen protozoa. *Nature* **1959**, *184(Suppl 12)*, 901–902.
37. Kim, H.; Chin, J.; Choi, H.; Baek, K.; Lee, T.-G.; Park, S.E.; Wang, W.; Hahn, D.; Yang, I.; Lee, J. Phosphoiodyns A and B, unique phosphorus-containing iodinated polyacetylenes from a *Korean sponge Placospongia* sp. *Org Lett.* **2013**, *15*, 100–103.
38. La Nauze, J.M.; Rosenberg, H. The identification of 2-phosphonoacetaldehyde as an intermediate in the degradation of 2-aminoethylphosphonate by *Bacillus cereus*. *Biochim Biophys Acta.* **1968**, *165*, 438–447.
39. Kittredge, J.; Isbell, A.; Hughes, R. Isolation and characterization of the N-methyl derivatives of 2-aminoethylphosphonic acid from the sea anemone, Anthopleura xanthogrammica. *Biochemistry* **1967**, *6*, 289–295.
40. Hidaka, T.; Imai, S.; Hara, O.; Anzai, H.; Murakami, T.; Nagaoka, K.; Seto, H. Carboxyphosphonoenolpyruvate phosphonomutase, a novel enzyme catalyzing CP bond formation. *J Bacteriol.* **1990**, *172*, 3066–3072.
41. Simon, M.A.; Ramos-Figueroa, J.S.; Reyes Lopez, V.; Ongpipattanakul, C.; Zhu, L.; Giurgiu, C.; Hoffpauir, Z.A.; Lamb, A.L.; Nair, S.K.; van der Donk, W.A. Discovery of the phosphonate flavophos produced by *Burkholderia*. *J Am Chem Soc.* 2026, 148, 18030 – 18043.
